# Multiracial Reading the Mind in the Eyes Test (MRMET): An inclusive version of an influential measure

**DOI:** 10.3758/s13428-023-02323-x

**Published:** 2024-04-17

**Authors:** Heesu Ally Kim, Jasmine Kaduthodil, Roger W Strong, Laura T Germine, Sarah Cohan, Jeremy B Wilmer

**Affiliations:** 1 Department of Neuroscience, Wellesley College, Wellesley, MA USA; 2 Division of Depression and Anxiety Disorders, McLean Hospital, Belmont, MA USA; 3 Institute for Technology in Psychiatry, McLean Hospital, Belmont, MA USA; 4 Department of Neurosciences and Shiley-Marcos Alzheimer’s Disease Research Center, University of California San Diego School of Medicine, La Jolla, CA USA; 5 Department of Psychiatry, Harvard Medical School, Belmont, MA USA; 6 The Many Brains Project, Belmont, MA USA; 7 Department of Population Medicine, Harvard Medical School and Harvard Pilgrim Health Care Institute, Boston, MA USA; 8 Department of Psychology, Wellesley College, Wellesley, MA USA

**Keywords:** Social cognition, Autism, Cognitive assessment, Diversity, Multiracial test, RDoC

## Abstract

Can an inclusive test of face cognition meet or exceed the psychometric properties of a prominent less inclusive test? Here, we norm and validate an updated version of the influential Reading the Mind in the Eyes Test (RMET), a clinically significant neuropsychiatric paradigm that has long been used to assess theory of mind and social cognition. Unlike the RMET, our Multiracial Reading the Mind in the Eyes Test (MRMET) incorporates racially inclusive stimuli, nongendered answer choices, ground-truth referenced answers, and more accessible vocabulary. We show, via a series of large datasets, that the MRMET meets or exceeds RMET across major psychometric indices. Moreover, the reliable signal captured by the two tests is statistically indistinguishable, evidence for full interchangeability. We thus present the MRMET as a high-quality, inclusive, normed and validated alternative to the RMET, and as a case in point that inclusivity in psychometric tests of face cognition is an achievable aim. The MRMET test and our normative and validation data sets are openly available under a CC-BY-SA 4.0 license at osf.io/ahq6n.

**Supplementary Information:**

The online version contains supplementary material available at 10.3758/s13428-023-02323-x.

## Introduction

At the broadest level, this paper aims to demonstrate that inclusiveness in psychological measurement is an achievable aim, and that this is true even in the domain of face processing, where, plausibly, inclusiveness might conflict with experimental control. We do this by developing, via a systematic, data-driven approach, an alternate, more race- and gender-inclusive, version of one of psychology’s most widely used tests, the Reading the Mind in the Eyes Test (RMET, Baron-Cohen et al., 2001b). We then test our new version, head-to-head, against the original. Conceivably, desirable characteristics of the original test—such as its reliability, convergent validity, discriminant validity, range of variation, or practical correlates (see Wilmer et al., 2012; Wilmer, 2017)—could be difficult to reproduce. This might be true even without inclusiveness as an added requirement, but especially with it. In a series of investigations, with large, diverse participant samples, we systematically investigate each of the abovementioned desirable characteristics. In each case, our new, more inclusive test meets or exceeds the original test. We present the new test as a proof of concept that inclusivity need not conflict with measurement quality, even in a domain such as face processing.

### Generality versus control in science: A necessary trade-off?

Merriam-Webster defines science as the search for “general truths or...general laws” (Merriam-Webster, 2020). The bedrock principle underlying generality is representativeness. By being representative of a larger population of interest, each part of the study design—the stimuli, responses, participants, manipulations, contexts—can establish generality. The present investigation is part of a broader effort to increase the representativeness of stimuli, responses, and participants in the human behavioral sciences (Dhami et al.,, 2004; Henrich et al., 2010).

The domain of the present work is social cognition, and our particular focus is on face reading: that is, reading the expression on another person's face. In face-reading research, a classic debate concerns the degree to which faces are read similarly across races, ethnicities, and cultures, and existing evidence points to important differences (Elfenbein & Ambady, 2002; Barrett et al., 2019). Such evidence suggests that incorporation of diverse and inclusive stimuli could add to the generality of what is measured by a face-reading test. Here, we will do just that: take a popular and influential face-reading measure and develop a high-quality alternative with more racially inclusive face stimuli.

Representativeness, however, is not the only scientific principle to consider in the design of human behavioral research studies. Another core principle is scientific control: the practice of systematically limiting potential sources of variation in order to isolate core mechanisms of interest. Herein lies a central question: to what degree do representativeness and scientific control directly trade off against each other? More specifically, does increased diversity and inclusion in stimulus selection increase the noise of measurement and/or reduce the validity with which certain mechanisms of interest can be isolated? Our experience suggests that these are not merely theoretical questions. Rather, they are considerations that commonly influence the design of studies in the field of social cognition. Because such questions typically remain unanswered, study design may, in the name of experimental control, be explicitly or implicitly nudged toward the use of less diverse and inclusive stimuli. Perhaps this is why, of the 14 major tests of face expression reading reviewed by Palermo and colleagues (Palermo et al., 2013), only one incorporated non-White stimuli.

To avoid singling out others for what is a widespread phenomenon, we present an illustrative example from our own published research. In a study of first impressions of trustworthiness in faces, we aspired toward representativeness by randomly selecting face stimuli from a large database with varied camera angles, lighting, picture quality, hairstyles, and makeup (Sutherland et al., 2020). However, we removed non-White faces. Our reasoning, at the time, was that since our recruitment was constrained to a population predominantly of European descent (via Twin Research Australia), it made sense to "avoid well-known other-race effects in face perception, which were not our focus" (Adams et al., 2010). In retrospect, this decision left unanswered the question of whether our results would generalize to the perception of non-White faces. The decision to exclude racially diverse stimuli is sufficiently common in the literature that it spurred a recent paper entitled “Why is the literature on first impressions so focused on White faces?” (Cook & Over, 2021).

### The RMET: Usage, validity, and development

**Fig. 1:**
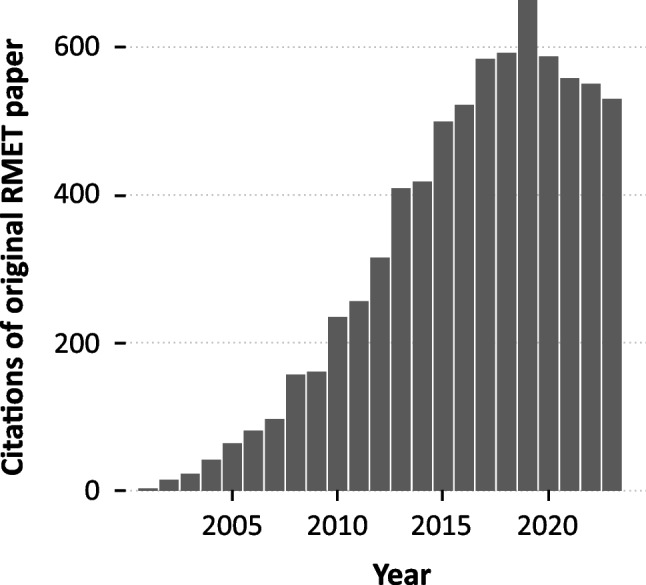
The Reading the Mind in the Eyes Test is gaining momentum in scholarly research. Citations of the paper that introduced the RMET (Baron-Cohen et al., 2001b) from the year 2002 until year 2021, based on Google Scholar citation metrics retrieved 8/17/2022

In the present research, we poured our efforts and resources into the creation of a new, more racially inclusive version of a particularly popular and influential test of face reading: the Reading the Mind in the Eyes Test (RMET; Baron-Cohen et al., 2001b). The RMET has been widely used in studies of clinical syndromes such as autism (Peñuelas-Calvo et al., 2019), schizophrenia (Bora et al., 2009), depression (Simon et al., 2019), borderline personality disorder (Fertuck et al., 2009), and Alzheimer’s disease (Yi et al., 2020). Further, it has also been widely used in studies of nonclinical adult populations on topics ranging from collective intelligence (Wooley et al., 2015), to brain function (Lizcano-Cortés et al., 2021), to response to stress and adversity (Germine et al., 2015), to development and aging (Hartshorne & Germine, 2015). The RMET was one of two tests recently recommended by the United States National Institute of Mental Health (NIMH) for assessing the understanding of mental states (NAMHC Workgroup, 2016). In total, the paper that introduced the RMET has been cited more than 7400 times (Baron-Cohen et al., 2001b). Contrary to the citation rate for most published work, which peaks soon after publication (on average after 4 years; Walters, 2011), the citation-rate for this paper increased for its first 18 years straight, with more than an order of magnitude more citations in its latest six years (3,481) than its first six (322) (Fig. 1, Google Scholar).

The RMET was originally introduced, in 1997, to address a key weakness in the literature on theory of mind (ToM): existing tests worked well only for small children (Baron-Cohen et al., 1997). For older children and adults, these tests were too easy; performance bunched up near the ceiling, making it difficult or impossible to distinguish normal from abnormal performance, except for the most severe impairments (Baron-Cohen et al., 1997). The RMET solved this problem in an unexpected manner: via a face expression reading test. This solution was unexpected because the measurement of face expression reading, like ToM, has long been plagued by ceiling effects (Palermo et al., 2013). Such ceiling effects are caused by the salience, and resulting ease of detection, for commonly studied emotional expressions—for example the upturned lips of happiness (Rutter et al., 2019). The RMET pulled performance off the ceiling via two clever manipulations: first, it restricted the view to the eye region only, and second, it dropped the “basic human emotions”—happiness, fear, sadness, disgust, surprise, and anger—in favor of more “complex…mental states” such as “reflective” and “scheming” (Baron-Cohen et al., 1997). A later, revised version of the RMET offered further improvements: an expanded range due to more items and more response options per item, and a new validation study that demonstrated correlations with a self-report measure of autistic-like traits (the Autism Spectrum Quotient, ASQ; Baron-Cohen et al., 2001a) and that described performance in a sizable normative sample of 239 participants (Baron-Cohen et al., 2001b).

To this day, the RMET remains a rare example of a test of either ToM or face expression reading whose range (lack of ceiling effect), reliability, and validity supports its use to capture the breadth of human variation in adults (Palermo et al., 2013; Dodell-Feder et al., 2020; Rutter et al., 2019); hence its continued wide usage. While this wide usage and resulting scientific influence reflects undeniable strengths of the RMET as a measurement instrument, that same wide usage increasingly highlights limitations in its original stimulus set. It is these limitations that our investigation seeks to address.

### The present investigation

**Fig. 2:**
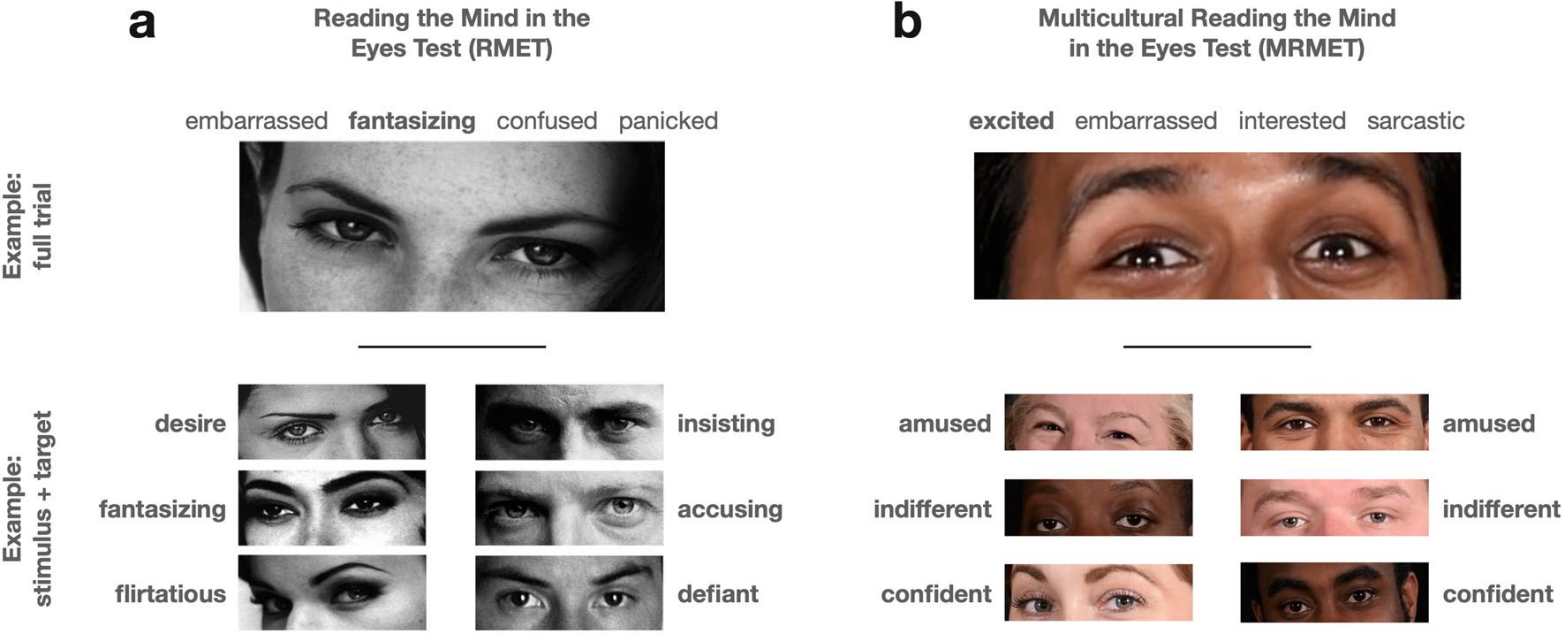
Example stimuli and responses for the Reading the Mind in the Eyes Test (RMET) and the Multiracial Reading the Mind in the Eyes Test (MRMET). (Top) For each of (**a**) RMET and (**b**) MRMET, one full trial is shown, with a face stimulus and its four response-word options (the target word is in **bold**). (Bottom) For each test, six illustrative face stimuli, three female and three male, and their target responses, are shown. RMET stimuli and responses show racial homogeneity (all European) and gender stereotyping (female sexualized, male assertive) relative to the MRMET’s racial diversity and less-gender-stereotyped images and target words

The stimuli in the RMET are eyes from faces, cut from magazines, and the task is to select one word out of four that best describes what the person is thinking (Baron-Cohen et al., 2001b). For present purposes, we focus on four notable characteristics of the RMET that are illustrated by Fig. 2a. First, the stimuli are exclusively white Eurocentric faces; no other racial or ethnic groups are represented. Second, both stimuli and responses play on gender stereotypes. Female faces are heavily made up, with dark eyeshadow and penciled-in eyebrows. Target responses for female but not male faces include sexualized language (desire, fantasizing, flirtatious), and target responses for male but not female faces include assertive language (insisting, accusing, defiant) (Fig. 2a; see Supp Table 1 for all response options). We have heard repeatedly over time from participants that they dislike the racial homogeneity and gender stereotyping of the RMET (Supp Table 2). Third, because the RMET’s stimuli were cut from magazines, there is no known ground truth for correct answers. That is, we do not know what thoughts were going through the minds of the persons who made the facial expressions. Fourth, the response options for the RMET include relatively sophisticated words (see Supp Table 1 for all response options).

Shown in Fig. 2b are a selection of stimuli and responses from our new test, called the Multiracial Reading the Mind in the Eyes Test, or MRMET. Unlike the RMET, the MRMET consists of racially inclusive stimulus faces. It utilizes non-gender-stereotyped stimuli and responses (see Table 1 for all response options). It incorporates a ground truth for correct answers: all expressions were produced by trained actors prompted with the desired target word. And it constrains response options to be relatively simple words (see Supp Table 1 for all response options). While the MRMET is not the first race/ethnicity-conscious alternative to the RMET—prior efforts produced Black (Handley et al., 2019) and Asian (Adams et al., 2010) versions of the RMET—we believe it is the first non-racially homogeneous version; the first version to receive direct, head-to-head, multivariate validation against the original RMET; and the first whose interpretation receives the rich contextualization provided by a large, diverse, normative data set.

**Table 1:**
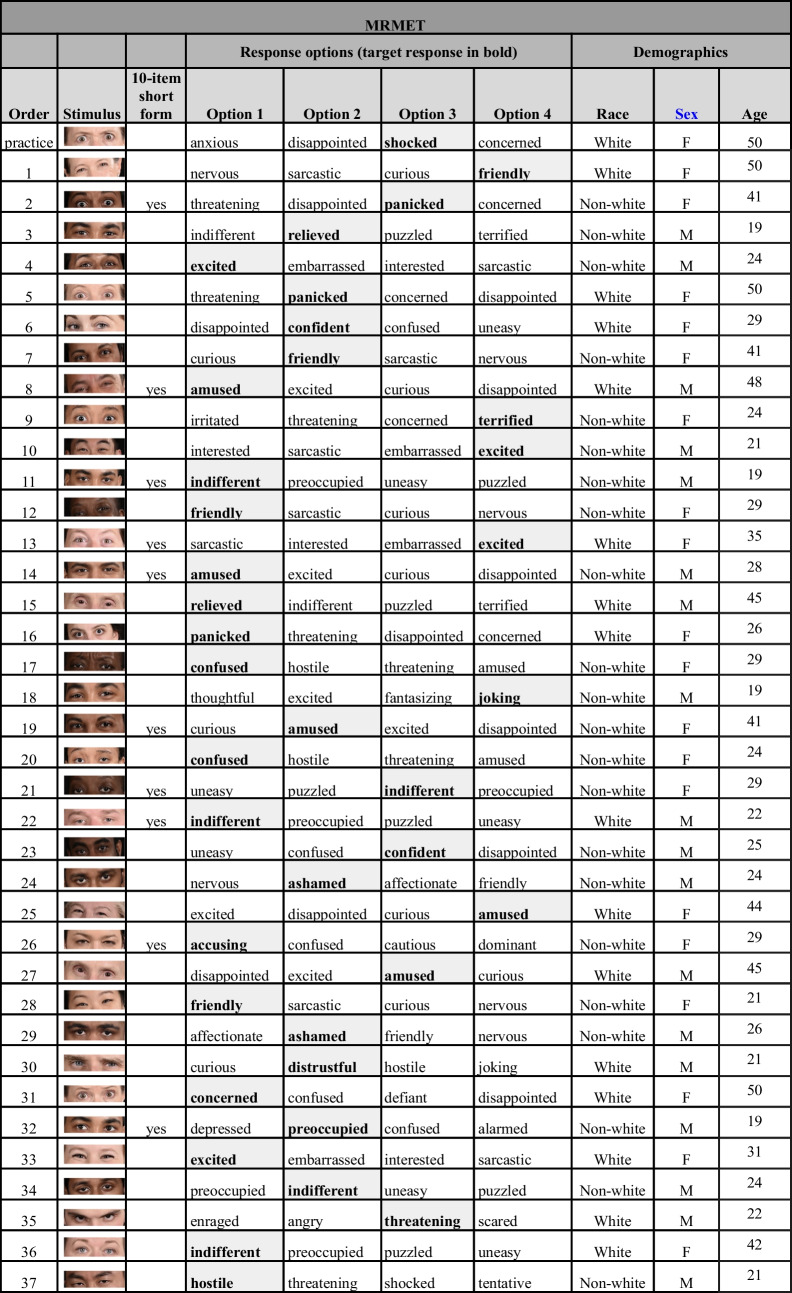
MRMET stimulus word choices and demographic information. Items for our suggested 10-item short form are indicated in the “10-item short form” column

Below, in “Results,” we show that the MRMET is psychometrically interchangeable with the RMET, and we present evidence for MRMET’s precision and validity. In “Methods,” we describe the process of developing the MRMET; we introduce large, open normative data sets, for both the MRMET and the RMET, to increase the capacity for psychometrically rigorous and clearly contextualized interpretation of future results using either test; and we suggest a reliable 10-item version of the MRMET for use in time-limited situations.

As was done for the RMET, we offer the MRMET as a free and open resource for research use. More broadly, we present the MRMET as a case in point that no hard and fast trade-off exists between representativeness and experimental control in the design of socially relevant tests using face stimuli. A decision can be made to develop a more representative test—both more racially inclusive and less gender-stereotyped—without cost to either its precision or its validity.

## Methods

### Initial MRMET item pool

Our goal was to create a new version of the RMET with racially inclusive stimuli, nongendered answer choices, ground-truth-referenced answers, and more accessible vocabulary. Stimulus images were derived from videos of racially and age-diverse actors expressing different emotions, taken as part of the Act Out for Brain Health project. We recruited professional actors from across a range of ages and races from the Boston theatre community. The actors were given mental state words to depict via their facial expressions and were recorded while holding a sheet of paper with the word they were depicting. Each actor depicted all the mental state words that served as target or non-target response options in the RMET. Individual images for each word were extracted from the video recordings when the expression was judged to have peaked, cropped to include eyes and eyebrows, and compiled into a database.

Once the face bank was created, we selected candidate images whose target (“correct”) response—that is, the word that the actor viewed to generate their expression—met two criteria. The first criterion was to avoid target responses such as “fantasizing” and “flirtatious” that were clearly gendered (see Fig. 2). The second criterion was to avoid target responses above a fifth-grade reading level, such as “baffled,” in favor of target responses at or below a fifth-grade reading level, such as “confused.” We then selected, from among the words used in the original RMET, three distractor (“incorrect”) response words per stimulus, based upon the same criteria. We ended up with 109 candidate items, each with one target response word and three distractor response words. All target and distractor words had shown up in the original RMET; and the target word was the word that the actor used to generate their face expression.

### Iterative development of the MRMET

From our initial stimulus pool of 109 candidate items, we selected 37 to comprise the MRMET (note that the MRMET is one item longer than the RMET, which has 36 items). We selected items for the MRMET based on several criteria. The first criterion was strength of correlation with a shortened, 16-item version of the RMET. This short version was produced by first computing the first principal component of the items in the RMET in a large pre-existing data set, then selecting the 16 items that correlated most highly with that principal component (item nos. 8, 9, 10, 11, 14, 15, 18, 19, 22, 24, 28, 32, 33, 34, 35, and 36). The items in this short version overlapped nearly completely with an independently derived 10-item shortened version of the RMET (Olderbak et al., 2015), including nine of that version’s 10 items (the 10 items are 8, 9, 12, 14, 15, 19, 22, 24, 32, and 36). We administered a version of the RMET that began with the 16-item short version and then presented a random set of 20 candidate items from the pool of 109 new items. Over 8000 participants completed the test, with an average of more than 1400 participants per item.

In addition to prioritizing items that correlated highly with the short RMET, we selected items based on four additional aims. First, we verified convergence between target word and consensus judgment. Second, we intentionally selected items whose face stimuli varied widely in racial and ethnic features, skin color, and age, and whose target responses varied widely in valence and arousal (Barrett et al., 2019). Third, we verified correlation between each item and the percent correct score on the rest of the items (of the 37 selected items, one had its target response revised to enhance this correlation). Fourth, in line with modern psychometric recommendations, we selected items with careful attention to item difficulty (Wilmer et al., 2012). Difficulty was defined as the percentage of persons who answered the item incorrectly. To facilitate optimal sensitivity across the full range of performance levels, we included items whose difficulties varied widely, from near-perfect (perfect difficulty = 0) to near-chance (chance difficulty = 75%, given the four response options); whose distribution was approximately normal, and whose mean, median, and mode were near the psychometric sweet spot midway between perfect and chance performance (difficulty = 37.5%) (Wilmer et al., 2012). If a participant took longer than 30 seconds to answer an item, they were told they were taking too long and were then given another 30 seconds to respond. The final product was a psychometrically data-honed test that contains multiracial, age-diverse, ground-truth-linked, non-gender-stereotyped faces and accessible response options across a range of facial expressions.

### Other measures

Apart from the RMET and MRMET, we employed three other tests: (1) a five-item Vocabulary test that asks participants to choose the closest synonym from among five options (this test uses the same format as TMB Vocabulary, Passell et al., 2019, Hartshorne & Germine, 2015; target words for the five items are swarm, despot, wily, aberration, mores), (2) a 90-second TMB Digit Symbol Matching (DSM) task (Passell et al., 2019; Chaytor et al., 2021) that is conceptually similar to the Wechsler Adult Intelligence Scale’s 90-second digit symbol coding test (Wechsler, 2008), and (3) an Autism Spectrum Communication (ASC) questionnaire that consists of a subset of eight communication-related questions from the widely used Autism Spectrum Quotient (ASQ) questionnaire (Baron-Cohen et al., 2001a; item nos. 20, 27, 31, 35, 36, 39, 45, and 48 from the ASQ, as recommended by English et al., 2020). Self-reported demographic variables (age, sex, education, and ethnicity) of the participants were also recorded.

The Vocabulary and DSM tests were administered to assess the MRMET’s discriminant validity, that is, its capacity to dissociate, to a similar degree as RMET, from non-face-reading tests. Given the MRMET’s focus on accessible response-word options, we predicted it would dissociate somewhat more strongly from vocabulary than the RMET does. The ASC was administered to assess the MRMET’s predictive validity, that is, its capacity to correlate, to a similar degree as RMET, with a key construct of interest: social skills that are reduced in autistic spectrum disorder. Demographic variables were collected to ask whether the MRMET would mirror the RMET’s patterns of performance across these demographic variables.

### Test reliabilities

Internal reliabilities of the tests in this study, computed if possible as Cronbach’s alpha (“alpha”), or, if not, as Spearman–Brown corrected split-half reliability (“split-half”), were as follows: RMET (alpha = .739, *n* = 17,730); MRMET (alpha = .711, *n* = 9295); five-item Vocabulary (alpha = .358, *n* = 1029); TMB DSM (split-half = .964, *n* = 2515); and ASC (alpha = .743 , *n* = 2515). MRMET, RMET, and Vocabulary are scored as percent correct. DSM is scored as the rate of correct responses (average correct responses per second, multiplied by proportion correct to penalize fast random responding). ASC is scored as the cumulative sum of a Likert scale self-rating (with higher scores better, after reversing the coding of negatively worded questions).

### Participant recruitment

All participants volunteered for this research through TestMyBrain.org, an internet-based citizen science research platform. TestMyBrain offers free, high-quality tests and provides immediate feedback on results relative to the broader population. Data from TestMyBrain have shown high quality—comparable performance levels, similar patterns of performance, and strong reliability and validity—when compared to traditional laboratory testing (Germine et al., 2012; Wilmer et al., 2010; Wilmer et al., 2012). For example, Germine and colleagues (Germine et al., 2012) found slightly (0.8%) higher mean RMET performance in TestMyBrain participants than in age- and sex-matched lab-tested participants. A similar comparison can be made between the large normative RMET data set in our present investigation (gleaned from TestMyBrain) and the original validation study for the RMET (where testing was done in a lab; Baron-Cohen et al., 2001b). The original RMET validation study found mean performance of 72.8% in a 122-person community sample whose mean age was 46.5 years. In our normative sample, we can achieve a similar mean age of 46.0 years by focusing on the 4352 participants who were over the age of 32, in which case mean performance is 74.0%. The demographic diversity of TestMyBrain volunteers is high relative to many traditional lab and online testing recruitment mechanisms (Wilmer et al., 2012). Before participating, all subjects provided informed consent according to the guidelines set by the Committee on the Use of Human Subjects at Harvard University.

### Data sets

The research reported here is based on three large data sets collected via TestMyBrain. These data sets include all participants who reported an age of 12–89 years. Two of these data sets, one each for the MRMET (*n* = 9295) and the RMET (*n* = 17,690), serve as normative data sets. That is, they enable measured patterns of performance across key demographic variables of interest. Such normative data can be used to contextualize scores; for example, a 15-year-old can be compared to other 15-year-olds. Basic characteristics of these normative data sets are shown in Table 2. We have published each of these data sets in an open format so that they can be maximally useful to future researchers. In addition to summary test scores, we include item-by-item data as well. Such item-level data can be used to dig deeper into item-level effects or to further refine the understanding of test performance, for example via item analyses or item response theory (IRT) analyses (Wilmer et al., 2012).

**Table 2:** Data sets used in this research

Demographic characteristics	RMET normative data set	MRMET normative data set	RMET + MRMET validation data set
Demographic characteristics			
Age — 25th / 50th / 75th percentiles No. who gave age	19 / 24 / 34 (17,626)	18 / 24 / 36 (9295)	18 / 23 / 34 (2515)
Gender — % female No. female / (no. female + male)	59.1% (10,267 / 17,362)	62.0% (5505 / 8876)	64.4% (1559 / 2422)
Education — % bachelor’s degree No. with plausibly terminal bachelor’s or above/all plausibly terminal degrees	54.0% (4919 / 9109)	50.5% (2585 / 5114)	56.9% (624 / 1096)
Ethnicity — % European No. European / (no. European + non-European)	75.7% (6829 / 9017)	85.2% (4232 / 4967)	77.2% (1099 / 1543)
Other information			
Sample size(*N*)	17,680	9295	2515
Years collected	2012 to 2015	2020 to 2021	2020

In addition to the MRMET and RMET normative data sets, we collected a third data set designed to directly compare the validity of the MRMET to that of the RMET. Table 2 shows the basic characteristics of this data set.

The most direct validity check was an assessment of interchangeability between the two tests. This was accomplished by splitting the tests in half. Participants either completed the first and second half from the same test (either the full MRMET or the full RMET; *n* = 1486) or completed half of each test (either first half MRMET and second half RMET, or first half RMET and second half MRMET; *n* = 1029). To the extent that half of the MRMET correlates with half of the RMET, both tests tap similar mechanisms. In the extreme, if these between-test correlations approach the correlation between the two halves of a single test, the mechanisms tapped are not only similar, but identical (Wilmer et al., 2012). Two tests that capture highly similar or identical mechanisms are interchangeable.

Additional validity checks were accomplished by comparing the MRMET’s and RMET’s correlations with the brief measures of three key constructs described above: vocabulary (Vocabulary test), processing speed (DSM), and autism-associated social skills (ASC). We computed correlations with these three measures at the level of MRMET and RMET half-tests. This analytic approach had two key advantages. First, it maximized sample sizes, and thus enhanced precision. As an illustrative example, consider that all participants who took the MRMET’s first half could be combined, regardless of whether they took the MRMET’s or RMET’s second half. Second, the approach of analyzing half-tests provided an internal replication check for analyses, because they were each conducted twice, once for each half-test.

### Reliability and validity analyses

In the reliability and validity analyses below, we use Spearman rank-order correlations for their robustness to outliers. All conclusions stay the same when Pearson correlations are used. This can be verified directly by observing the lack of major outliers in the scatterplots or by analyzing the open data sets. As a further verification of the robustness of results to varied analytic choices, the scatterplots for Figs. 5, 6, and 7 are drawn such that the physical slope of the regression line shown is equal to the Pearson correlation coefficient.

### Demographic variables and inclusion criteria

Figure 4 shows demographic analyses of age, gender, education, and ethnicity via the open normative data sets for the RMET and the MRMET, respectively. Table 2 shows demographic counts for these two data sets and for the third data set that administered halves of the RMET and MRMET (the “half-test” data set). These three open data sets include all recruited participants except those who specified an age outside the range of 12–89, regardless of their response to the gender, education, and ethnicity questions. For the analyses and counts of each of the four demographic variables, we included participants regardless of their responses (or non-responses) to the other three. Further inclusionary criteria for analysis of individual demographic variables are as follows.

For the age analysis, we included all participants who specified an age of 12–89. For the RMET normative data set, the age question was optional and 54 of 17,690 participants (0.03%) chose not to answer it. For the other two data sets, the age question was required.

For the gender analysis, a complication was that nonbinary gender had been handled differently for the two normative data sets. For the MRMET normative data set (and the half-test data set), the gender question was required and provided the option of “nonbinary or genderqueer,” which was selected by 419 of 9295 participants (4.5%). For the RMET normative data set, the gender question was not required and did not offer an explicit nonbinary option, and 381 of 17,690 participants (1.8%) chose not to answer as either male or female. For the counts in Table 2, we include nonbinary individuals in the denominator for the MRMET but exclude nonresponders from the denominator for the RMET because of their unknown gender. For the graphs in Fig. 4, we plot only male and female responses because these responses are directly comparable across the two tests.

For the education analysis, to reduce the potential confounding influence of age, we first regressed out age and age-squared (a quadratic fit that fit the data well; see Fig. 4 and its legend). We then filtered out younger ages to exclude either those who were too young to have likely achieved the reported level of education by the reported age (e.g. a bachelor’s degree at age 12), or those who were sufficiently young that they could still achieve the next higher level of education at a typical or modal age (e.g., those who reported some college and were still younger than age 22, the modal age for receiving a bachelor’s degree). Specifically, we excluded those reporting a high school degree or less who were younger than 20, those reporting some college (including an associate degree) who were younger than 22, those who reported a bachelor’s degree who were younger than 24, and those reporting a graduate degree who were younger than 26.

For the ethnicity analysis, to reduce the potentially confounding influence of non-native English skills, we included only individuals who responded from a country where English was the majority language. These majority-English-speaking countries are (along with *N* for RMET normative data set, then *N* for MRMET normative data set) the United States (6403, 3946), Great Britain (1533, 1002), Canada (1062, 515), Australia (542, 370), New Zealand (121, 65), Ireland (78, 63), Trinidad and Tobago (11, 7), Jamaica (9, 4), the Bahamas (2, 2), Belize (1, 2), Barbados (2, 1), Saint Vincent and the Grenadines (2, 0), Grenada (1, 1), Guyana (1, 0), Antigua and Barbuda (0, 1), Saint Lucia (1, 0), Dominica (0, 0), and Saint Kitts and Nevis (0, 0).

While there was some judgment involved in these inclusionary criteria, alternate approaches produced similar results. Part of the reason we have kept these normative data sets relatively unfiltered, and made them openly available, is to enable others to reanalyze these data in different ways.

### MRMET short form

For circumstances where testing time is limited, it can be helpful to have available a short form of a test. We recommend the following 10-item MRMET short form (items: 2, 8, 11, 13, 14, 19, 21, 22, 26, and 32). These 10 items were selected as those that correlated most highly, in the first half of our MRMET normative data set (*n* = 4648), with the first principal component computed on the 36 remaining items, excluding the one item (of 37 total) that was being assessed. The internal reliability of this short form (alpha = 0.59), computed on the second half of our MRMET normative data set (*n* = 4647), is similar to that of the RMET’s recommended 10-item short form (alpha = 0.57; Olderbak et al., 2015), computed on our RMET normative data set.

## Results

The aim of the present work was to provide an alternative to the Reading the Mind in the Eyes Test (RMET) that incorporated four key enhancements: racial inclusivity, nongendered answer choices, ground-truth-linked correct responses, and more accessible vocabulary. But is the RMET's success reproducible? Conceivably, there might be something uniquely effective about the RMET’s specific face stimuli or response options that is difficult to reproduce. Alternatively, even if new face stimuli and response options could theoretically work, one or more of our four enhancements might interfere with test effectiveness. In the worst case, there could be hard, broad trade-offs between representativeness and scientific control that strictly limit the enterprise of representative test creation.

In the sections below, we systematically contradict these notions of systematic trade-offs and lack of reproducibility in stimulus choice. We start by providing converging evidence from two separate analyses that the MRMET is essentially equivalent to, and therefore interchangeable with, the RMET. First, direct evidence for interchangeability comes from the correlations of MRMET and RMET with each other. Second, indirect evidence is seen in MRMET’s demographic associations, which mirror those of RMET. In a third section, we point out that MRMET shows a more optimal distribution of performance for precisely capturing performance across the full range, especially at the high end. In a fourth and fifth section, we complement the near-equivalence between MRMET and RMET with evidence for substantial nonequivalence, of both MRMET and RMET, with measures of processing speed and vocabulary. Such nonequivalence is demonstrated via dissociations that, if anything, are stronger for MRMET than for RMET. Finally, we examine the ASC, a survey instrument that captures an array of practically important social skills that are believed to be specifically impaired in autism spectrum disorder. Here, too, MRMET’s correlations with ASC mirror RMET’s, suggesting an equivalent level of social and/or clinical relevance. Together, these results establish the pattern of associations and dissociations that has long been the gold standard for establishing the validity of any new test (Cronbach & Meehl, 1955). We therefore present the MRMET as a valid, interchangeable alternative that can be used in place of the RMET.

### Interchangeability: MRMET captures the same signal as RMET

The most direct evidence of interchangeability between two tests is a substantial correlation between the two. In this context, it is instructive to think in terms of reliably measured variation. Scores on any measure are partly due to reliably measured variation (aka “signal”) and partly due to measurement error (aka “random variation,” or just “noise,” as with lucky versus unlucky guessing). The higher a test’s measurement error, the lower its reliably measured variation, and the less it is capable of correlating with another measure, or even with itself. Reliable variation is, in fact, estimated via one of several techniques that correlate the test with itself (Wilmer et al., 2012). In the extreme, if two measures share the exact same signal, then they should correlate with each other to the same degree as they correlate with themselves. That is, the reliability of the tests being correlated constitutes the theoretical ceiling—the maximum possible value—of their correlation with each other.

**Fig. 3:**
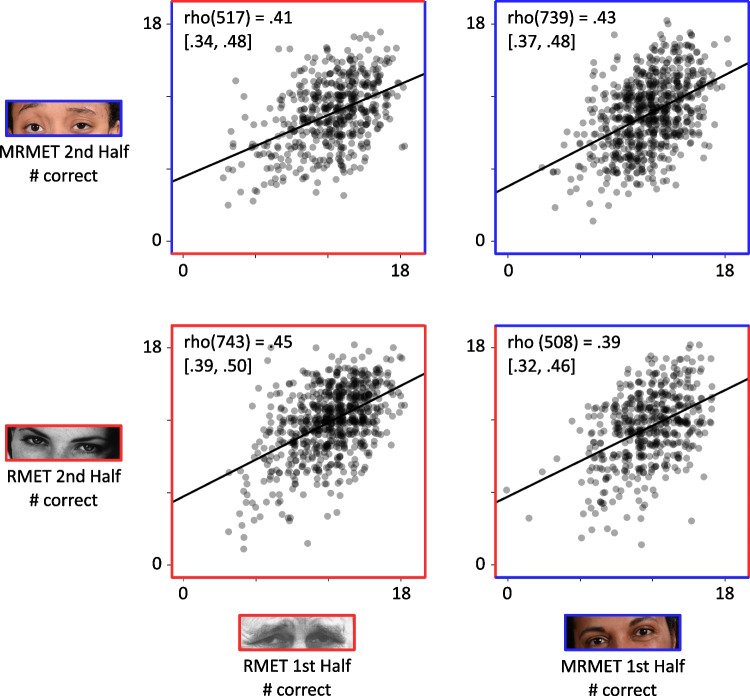
Between- and within-test correlations. MRMET and RMET are similar across test conditions. Each test was split into first and second halves; axes are labeled by half (first, second) and by test (RMET, MRMET). Scatterplots show associations between half-tests. Cross-test correlations are nearly equal to within-test correlations, evidence that the two tests capture essentially equivalent signal, and therefore could be used interchangeably. Shown are Spearman rho correlation values, which are used throughout this paper for their robustness to influential data points and outliers, but results remain highly similar with Pearson *r* correlation values. Lines are least-squares regression lines

A relatively concrete and tangible way to quantify a test’s reliability is to look at the correlation between its two halves. That is the approach we take here, though other approaches produce the same conclusions. In Fig. 3, the bottom-left graph shows the Spearman correlation, rho(743) = .45 [.39, .50], between the RMET’s two halves, and the top-right graph shows the Spearman correlation rho(739) = .43 [.37, .48] between the MRMET’s two halves (all correlations reported below are Spearman rho). The geometric mean between these two within-test correlations, which is .44, serves as the theoretical ceiling for the cross-test correlations, shown on the other diagonal of Fig. 3.

The top-left graph shows the rho(517) = .41 [.34, .48] correlation between the RMET’s first half and the MRMET’s second half, and the bottom-right graph shows the rho(508) = .39 [.32, .46] correlation between the MRMET’s first half and the RMET’s second half. These cross-test correlations are not only numerically similar to, but also statistically indistinguishable from, the two within-test correlations. That the cross-test correlations come this close to matching within-test correlations demonstrates essential equivalence between the RMET and the MRMET. That is, MRMET scores capture nearly all the signal that RMET scores capture, differing primarily due to the inevitable noisiness of measurement, not due to diverging signal. This exceptional degree of convergence between the two tests supports their interchangeability.

### Demographic mirroring: MRMET and RMET show parallel demographic patterns

While direct correlations between tests provide the most direct evidence for interchangeability, an independent source of information is provided by the tests’ correlations with demographic variables. To the extent that the MRMET shows demographic trends that parallel those for RMET, that would further support the interchangeability of MRMET and RMET in a domain with clear practical relevance. Specifically, we examine age, gender, education, and ethnicity in large normative data sets collected via TestMyBrain.org (see “Methods”).

**Fig. 4:**
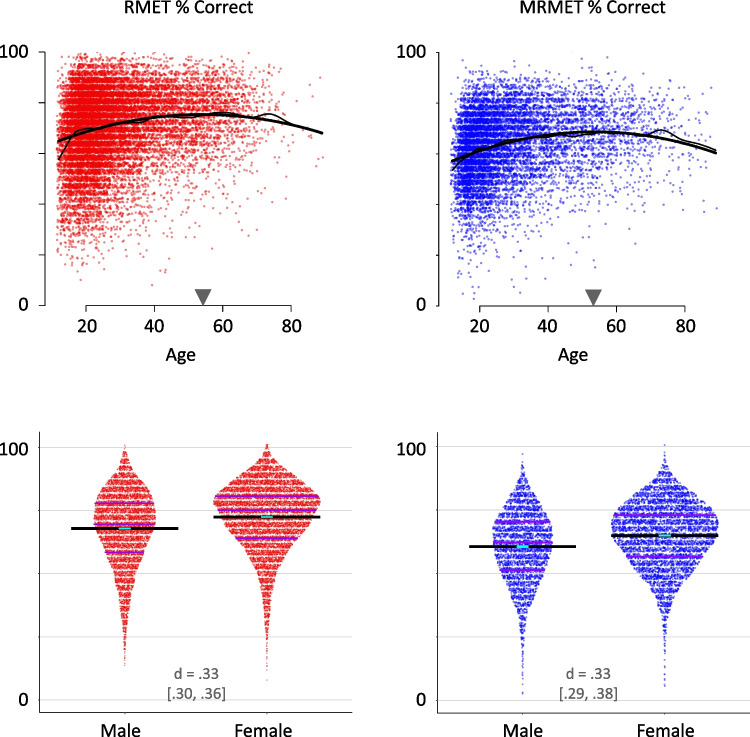

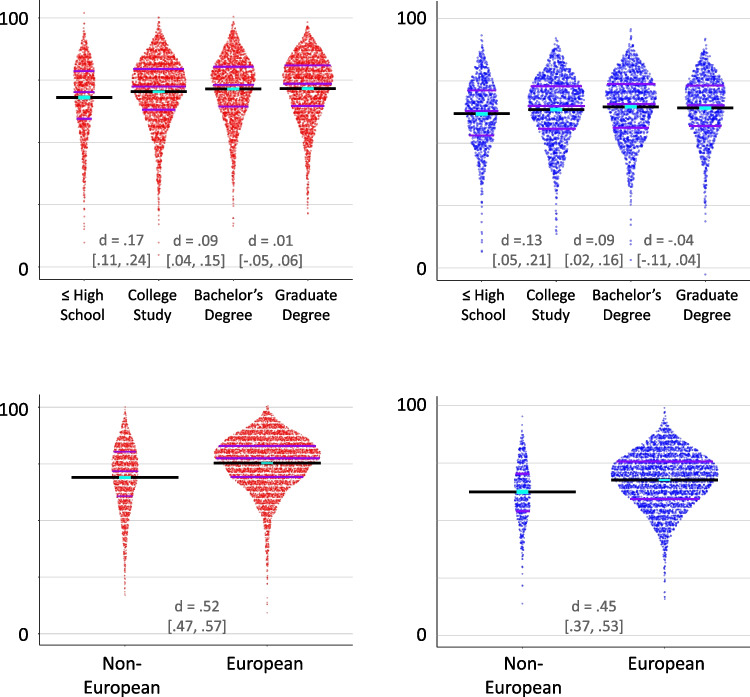
Patterns of score distributions across demographic subgroups are similar across both original RMET and MRMET. **Age (row 1):** Plots demonstrate trends across the lifespan for scores on the RMET (left) and MRMET (right). The dark curve, a quadratic fit, shows a relatively long plateau with late peaks (black triangles) in the 50s. The light curve, a spline fit, was computed via R’s smooth.spline function with spar smoothness parameter set to 40. The close correspondence between quadratic and spline fits supports the use of the simpler quadratic fit. **Gender (row 2):** Plots demonstrate higher scores for females on both RMET and MRMET, with the same effect size for both tests. **Education (row 3):** Plots demonstrate modest increases in performance with greater education. **Ethnicity (row 4):** Plots demonstrate higher scores in those with European ancestry. **Row 2–4 notes:** Cohen’s *d* values, with 95% CIs, are provided as a standardized effect size measure to quantify the differences between adjacent groups. Blue boxes show 95% CIs on individual group means. See “Methods” for the procedures we followed to reduce age confounds in the measurement of education and language confounds in the measurement of ethnicity. Plots used are sinaplots, which are similar to violin plots in that data density for a given *y*-value is indicated by sinaplot width. Unlike violin plots, however, sinaplots create the violin shape out of the data points themselves, so that individual data values can be seen and examined. Values in sinaplots are jittered vertically where needed to facilitate data point visibility. Purple lines indicate 25th, 50th, and 75th percentile scores

Our first analysis looks at age. Prior studies in large cross-sectional samples revealed two unusual features in RMET’s age curve (Hartshorne & Germine, 2015). First, it peaked relatively late, in the 40s or 50s. Second, it showed an unusually long and flat plateau in its peak performance between approximately age 40 and 60. As Fig. 4, row 1 shows, we replicate these features for both RMET and MRMET, with wide plateaus that peak in the 50s.

For the RMET, a gender difference favoring females over males has been considered a key indicator of its validity (Baron-Cohen et al., 2015). As Fig. 4, row 2 shows, we both replicate this gender difference with RMET (left; *d* = 0.33), and we find a MRMET gender difference that is identical in both direction and size to MRMET (right; *d* = 0.33).

The RMET has been shown to correlate with higher levels of education, potentially a result of the relatively complex language used for its response-word options (Dodell-Feder et al., 2020). As Fig. 4, row 3 shows, we find small but robust increases in performance from high school education through to a college bachelor’s degree for both RMET and MRMET, and no difference between bachelor’s and graduate degrees for either test. The increases are somewhat larger for the RMET than the MRMET, perhaps owing to its more complex vocabulary.

**Fig. 5:**
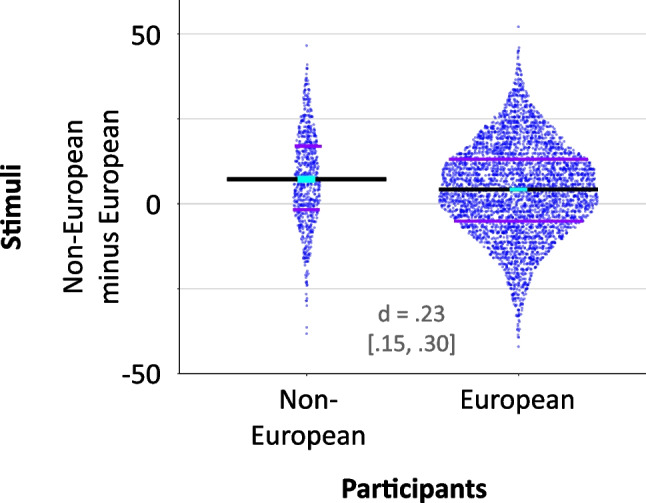
Ethnicity of the stimulus interacts with ethnicity of the participant. Participants who indicate a fully non-European ethnicity have a greater advantage (7.2% advantage, 95% CI [6.2%, 8.2%], *N* = 735) for non-European ethnicity stimuli over European ethnicity stimuli than participants who indicate a fully European ethnicity (4.2% advantage, 95% CI [3.8%, 4.6%], *N* = 4232; difference is 3.0%, 95% CI [2.0%, 4.1%]). The ethnicity difference between European and non-European participants for European stimuli is 7.1% (95% CI [6.0%, 8.2%]), and for non-European stimuli it is 4.1% (95% CI [3.1%, 5.1%]). For comparison, the ethnicity difference between European and non-European participants for the RMET (which uses exclusively European stimuli) is 6.35% (95% CI [5.8%, 6.9%])

The RMET has been shown to correlate with ethnicity such that persons of European descent obtain higher scores (Dodell-Feder et al., 2020). As Fig. 4, row 4 shows, we find a similar overall effect for both RMET and MRMET; however, an analysis of stimulus ethnicity (Fig. 5) in the MRMET shows that the effect is smaller for non-European stimuli than for European stimuli.

Together, the RMET’s and MRMET’s results for age, gender, education, and ethnicity parallel each other to such a degree that they strongly support the interchangeability of MRMET with RMET. That said, while close demographic mirroring contributes to the case for interchangeability, it is not in all cases desirable, especially with a variable like ethnicity, where one might reasonably hope to minimize differences. We discuss this as a limitation of the present work in the Discussion below.

### Sweet spot: MRMET shows optimal average performance and less skew

As can be seen from Fig. 4, MRMET had a lower mean score (63.4%) than RMET (70.5%). While neither test showed a severe ceiling effect, RMET’s was somewhat greater than MRMET’s, and therefore RMET showed a stronger negative skew (−0.75) than MRMET (−0.49). A mean score midway between perfect and chance performance is considered a psychometric sweet spot for reliably capturing the full range of performance (Wilmer et al., 2012). For a test like RMET or MRMET, with four response options, this sweet spot is 62.5%. The proximity of MRMET’s mean score to this sweet spot could enhance its capacity to distinguish scores at the high end of performance.

### Discriminant validity: MRMET dissociates from vocabulary as much or more so than RMET

In the first section above, the strength of MRMET’s association with RMET demonstrated a high degree of shared signal, and thus essential interchangeability. In the next two sections, we examine correlations with tests of capacities that differ from the face-reading capacity that both RMET and MRMET intend to measure. For these analyses, it is not the strength of association, but rather the strength of dissociation, that marks valid measurement. This type of dissociation-based test validation is typically referred to as a discriminant validity check (Wilmer et al., 2012).

The first test we examine is the five-item Vocabulary test (see “Methods”). While to some degree, language may be integral to the capacity for reading faces and for working out the intentions of others (Malle, 2002), one would still hope for a reasonable degree of dissociation between vocabulary per se and face reading. Indeed, the RMET has been specifically criticized for the complexity of its language (Dodell-Feder et al., 2020), which raises the possibility that some portion of the RMET’s signal could be driven by the raw strength of one’s vocabulary. As mentioned above in “Methods,” a key aim of ours in selecting items for the MRMET was to cut down on this language complexity to make the MRMET more accessible than the RMET.

**Fig. 6:**
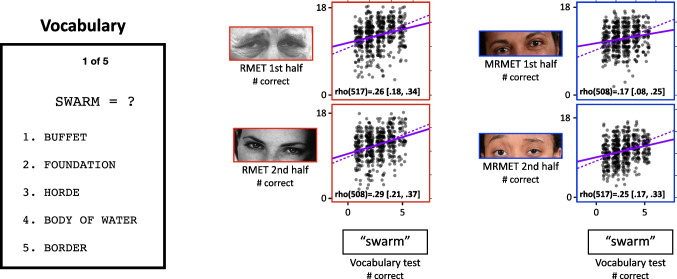
MRMET dissociates from vocabulary somewhat more strongly than RMET does. On the left is shown one trial from the five-item Vocabulary test. Participants were asked to identify which of the five words below was the closest synonym for the word at the top. In the middle is a pair of scatterplots showing the correlations of each RMET half-test with the Vocabulary test. On the right is a pair of scatterplots showing the correlations of each MRMET half-test with the Vocabulary test. Provided in each plot is the Spearman rho correlation with 95% CI. The solid purple line is the least-squares regression line, with axis ranges selected such that the physical slope of that line is equivalent to the Pearson correlation coefficient. The physical slope of the dashed purple line indicates the upper bound on the correlation value between these two measures

With these considerations in mind, we assessed the degree to which RMET and MRMET would dissociate from vocabulary. In Fig. 6, we can see that each half of the MRMET correlates numerically less with the Vocabulary test than does either half of the RMET. While these reductions are modest, they are in the direction one would hope for in a more language-accessible test. An important comparison when looking for dissociations is the computed upper bound on correlations between the two tests given their reliabilities (aka their correlations with themselves). This upper bound is computed as the geometric mean of the reliabilities of the two tests (Wilmer et al., 2012). Given the .36 reliability of the Vocabulary test and the respective .45 and .42 split-half correlations of RMET and MRMET (split-half correlations serve as an estimate of the reliability of each half-test), the upper bound for the correlations of RMET and MRMET with the Vocabulary test are .40 and .39, respectively. All four correlations shown in Fig. 6 are substantially below these values. Even the upper end of the 95% confidence intervals around the correlation values shown in Fig. 6 fall short of these upper bound values. Together, these results provide evidence that both RMET and MRMET dissociate at least partly from raw vocabulary.

**Fig. 7:**
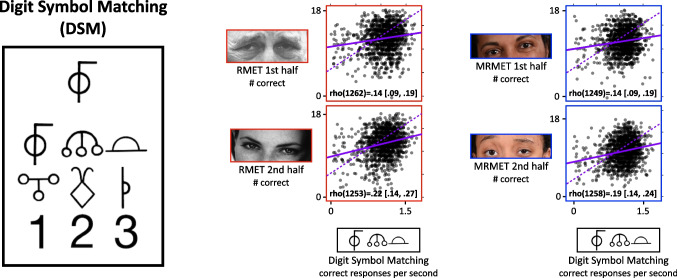
MRMET dissociates strongly from the TMB Digit Symbol Matching (DSM) test of processing speed. On the left is shown one trial from the DSM test. Participants were asked to press the key for the number corresponding to the target symbol that is presented at the top. The key was always present below the shown target symbol. Participants identified as many symbols as they could within 90 seconds. In the middle is a pair of scatterplots showing the correlations of each RMET half-test with the DSM test. On the right is a pair of scatterplots showing the correlations of each MRMET half-test with the DSM test. Provided in each plot is the Spearman rho correlation with 95% CI. The solid purple line is the least-squares regression line, with axis ranges selected such that the physical slope of that line is equivalent to the Pearson correlation coefficient. The physical slope of the dashed purple line indicates the upper bound on the correlation value between these two measures

**Fig. 8:**
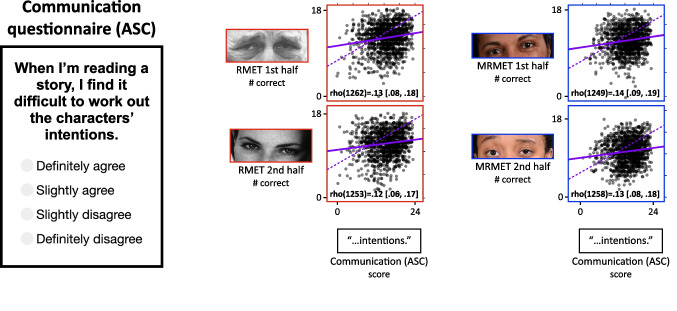
MRMET and RMET both show the expected modest but statistically robust associations with the Autism Spectrum Communication (ASC) questionnaire. On the left is shown one trial from the ASC instrument. Participants were asked to indicate their level of agreement with eight communication-related statements. In the middle is a pair of scatterplots showing the correlations of each RMET half-test with the ASC questionnaire. On the right is a pair of scatterplots showing the correlations of each MRMET half-test with the ASC questionnaire. Provided in each plot is the Spearman rho correlation with 95% CI. The solid purple line is the least-squares regression line, with axis ranges selected such that the physical slope of that line is equivalent to the Pearson correlation coefficient. The physical slope of the dashed purple line indicates the upper bound on the correlation value between these two measures

As a visual aid, we have selected axis ranges in Fig. 6 (and Figs. 7 and 8 as well) such that the physical slopes of the regression lines, shown in solid purple, exactly equal the Pearson correlation coefficients for the data. This then allowed us to also plot, as purple dashed lines, the slopes corresponding to the computed upper bounds on these correlations. The difference between the slopes of the solid and dashed lines can be interpreted directly as the degree of dissociation shown between the two tests.

### Discriminant validity: MRMET and RMET both dissociate strongly from processing speed

The second test we use to probe discriminant validity is the TMB Digit Symbol Matching (DSM) test (Passell et al., 2019; Chaytor et al., 2021), a measure of processing speed that was inspired by the digit symbol coding subtest of the Wechsler Adult Intelligence Scale (Wechsler, 2008). As processing speed is considered a core component of general cognitive ability (Carroll, 1993), and as it has shown relationships with social perception measures (Froiland & Davison, 2020), we considered it a good candidate for a study of the discriminant validity of MRMET and RMET.

As Fig. 7 shows, the correlations of both the RMET and MRMET with DSM were far lower than their respective upper bounds of .84 and .83, evidence for strong dissociations of both the RMET and MRMET from processing speed. Even the top ends of the 95% confidence intervals fell far short of these upper bounds. For both RMET and MRMET, the dissociation with DSM, as measured by the difference between the measured correlation and the upper bound, was stronger than the dissociation with Vocabulary. Together, these dissociations support the discriminant validity of both RMET and MRMET relative to processing speed.

### Social relevance: MRMET and RMET both correlate with self-reported communication skills

Much of the validity information for the RMET in prior work came from studies that showed lower performance in clinical syndromes such as autism (Peñuelas-Calvo et al., 2019), schizophrenia (Bora et al., 2009), depression (Simon et al., 2019), and Alzheimer’s disease (Yi et al., 2020). A consistent finding in nonclinical populations, however, has been the presence of a modest but statistically robust correlation between the RMET and the Autism Spectrum Quotient (ASQ). For example, across two studies with a total of 743 participants, this correlation averaged *r* = .13 (Baron-Cohen et al., 2015; Voracek & Dressler, 2006). The computed 95% CI for a .13 correlation with 743 participants is [.06, .20]. It is important to note that modestly sized correlations are the norm between performance-based tests like the RMET and self-report instruments like the ASQ. Such correlations are often modest even when the performance-based and self-report measures are designed to measure the exact same thing (Zell & Krizan, 2014). For example, a meta-analysis of the correlation between performance-based and self-reported memory in 24,897 persons found an average correlation of *r* = .15 (Beautoin & Desrichard, 2011).

Here, we assess the correlation of MRMET and RMET with the Autism Spectrum Communication (ASC) questionnaire, which is a subset of the ASQ. The ASC has emerged as a separate entity in factor analytic studies (English et al., 2020). Additionally, the ASC was identified in the original RMET validation study as a subtest that correlated with RMET in a nonclinical student sample (*r*(78) = .25 [0.03, 0.45]). We also confirmed in a pilot study that it appeared to show a modest but robust correlation with the RMET. As Fig. 8 shows, in the present sample, both halves of the RMET and both halves of the MRMET showed the expected modest but statistically robust correlation with the ASC. All four of these correlations were between .12 and .14, consistent with prior results (Baron-Cohen et al., 2015; Voracek & Dressler, 2006). Importantly, the correlations for the MRMET were not lower than those for the RMET, again suggesting that MRMET captures the same valid signal as the RMET.

## Discussion

Here, we report on the development and validation of a new inclusive version of the classic Reading the Mind in the Eyes Test (RMET), one of the most widely used measures of individual differences in face expression reading, mental state inferencing, and social cognitive ability (Baron-Cohen et al., 2001b). Based on analysis of data from a large and diverse sample totaling more than 10,000 participants, the Multiracial Reading the Mind in the Eyes Test (MRMET) shows the same or similar psychometric and demographic characteristics as the original RMET. The two tests had similar reliability, with correlations between tests so high as to indicate that they shared essentially all their reliable signal, evidence for full interchangeability. Scores on the two tests had similar associations with the demographic characteristics of age, gender, education, and ethnicity. Associations with scores on the Autism Spectrum Quotient were similar across both tests. Divergent validity (based on correlations with processing speed, measured via digit symbol matching performance) was also similar across both the original RMET and the MRMET. MRMET performance was somewhat less associated with vocabulary performance compared to RMET. Thus, the only hint of a psychometric difference between the two tests is a potentially lower reliance on vocabulary for the MRMET.

From a validation standpoint, the RMET and the MRMET likely measure the same underlying construct with similar precision. The correlation in performance between the MRMET and the RMET was at the ceiling for possible correlations based on the internal reliability of each test. Thus, the MRMET could be used in place of the RMET in many contexts—particularly those where the target population of participants is not homogeneously of European ancestry. While the RMET was designed for use in a relatively homogeneous population based in the UK (Baron-Cohen et al., 2001b), it has now become one of the most widely used measures of social cognition across the world. The success of the RMET thus became the reason for one of its most critical failings—a stimulus set that doesn’t represent the populations that the RMET is used to assess (Dodell-Feder et al., 2020). The development of the MRMET is our attempt to keep all that is useful about the RMET, but with stimuli that reflect the diversity of participants that the RMET is now used to assess.

One of the reasons the original RMET gained such broad use is the decision by the original creators to make the test widely available through the website of the University of Cambridge Autism Research Centre. Although broad distribution of stimuli might result in the overexposure of those stimuli, which could limit their utility in certain diagnostic and research settings, we believe that the advantages of accessibility and open science outweigh the disadvantages. Thus, we have created a package of materials distributed under an open-source license (CC-BY-SA 4.0) that will allow others to use the stimuli and test in their research studies. The test materials are provided at osf.io/ahq6n, and interested researchers may contact us to integrate web-based implementations of the MRMET into their studies. In addition to the full-length MRMET, we suggest a 10-item short form to use in time-limited circumstances (“Methods”); this short form compares favorably to a widely used 10-item short form of the RMET (Olderbak et al., 2015).

Importantly, feedback from our participants suggests that inclusiveness per se is an important step forward. Additionally, the higher scores that non-European individuals attained for non-European face stimuli, as compared with European face stimuli, clearly demonstrates that diverse stimuli can make a difference. Nonetheless, the reader will note that the overall association of performance with participant race/ethnicity and other demographic variables remains similar for the two tests (Fig. 4). Importantly, key goals here included the production of a culturally appropriate and more racially representative alternative to the RMET that captured a similar signal and exhibited no major loss in validity. In all these respects, we succeeded. Indeed, the signal was similar to the point of interchangeability, and the MRMET’s validity was high to the point of equaling or exceeding RMET’s. Arguably, it is not possible to both target high convergence with the RMET *and* show a different pattern of association with key demographic and individual differences variables. A critical remaining long-term aim, in our view, is to uncover the precise causes of demographic disparities across RMET, MRMET, and other social cognitive tests. Such understanding would allow those causes to be addressed head-on, whether they relate to testing approaches or to deeper societal inequities. In the meantime, the normative data sets we provide, for both MRMET and RMET, provide a valuable opportunity to carefully contextualize scores in a way that is mindful of existing demographic differences.

There are several limitations of the MRMET and our current validation work that should be considered. First, data collection was done entirely over the web. While we have previously found that web-based and lab-based administration of the RMET have similar psychometric characteristics (Germine et al., 2012), it is possible that factors related to the context of administration might impact scores. For example, participants might be more likely to look up the definitions of words in an unsupervised web-based setting than an in-person setting. Second, we also note that our analyses of the associations between test performance and sociodemographic characteristics were based on data collected over different time periods (data on convergent and divergent validity were collected at the same time). Thus, it is possible that patterns were distinct between the RMET and MRMET, but that these differences were offset by population-related changes in the association between performance and sociodemographic variables over time, making the two tests appear more similar. Third, the MRMET is not the only RMET-style test to incorporate non-White stimuli. Handley and colleagues (Handley et al., 2019) developed an RMET with the same target words and similar difficulty as the original RMET but with Black faces, and Adams and colleagues (Adams et al., 2010) did the same for Asian faces. While neither study reported reliability, validity, or normative data, such data could be collected in the future. Interestingly, the Asian RMET did, but the Black RMET did not, produce an interaction with race, in comparison with the original RMET, whereby people of the same race performed better and people of the opposite race performed worse (Handley et al., 2019; Adams et al. (2010).

## Conclusion

Our science can only be as good as our measures. Here, we have provided a case in point that the validity of an influential social cognitive measure, the RMET, was in no way tied to the non-inclusiveness of its stimuli. The MRMET demonstrated psychometric qualities that were as good as or better than the RMET, and it did so in large and demographically diverse samples. We hope that the development and validation of the MRMET leads us a step closer to the goal of a science that includes and represents the diversity of humans it exists to serve.

### Supplementary Information

Below is the link to the electronic supplementary material.

Supplementary file1 (DOCX 271 KB)

## Data Availability

The three datasets used in this study are made available on OSF (https://osf.io/ahq6n/).
