# Supplementary material for: Multiracial Reading the Mind in the Eyes Test (MRMET): An inclusive version of an influential measure
Source: Behav Res Methods. 2024 Apr 17;56(6):5900–17. doi: 10.3758/s13428-023-02323-x (PMC11335804; doi:10.3758/s13428-023-02323-x)
Supplement: Supplementary file 1 — Supplementary file1 (DOCX 271 KB) [file 13428_2023_2323_MOESM1_ESM.docx]

**SUPPLEMENTARY INFORMATION**

**Supplemental Table 1. RMET stimulus word choices and demographic information. Items for 10-item short form (Olderbak et al, 2015) are indicated in the “10-item short form” column.**

| **RMET** | | | | | | | | |
| --- | --- | --- | --- | --- | --- | --- | --- | --- |
|  |  |  | **Response options (target response in bold)** | | | | **Demographics**  **Ages approximated** | |
| **Order** | **Stimulus** | **10-item short form** | **Option 1** | **Option 2** | **Option 3** | **Option 4** | **Race** | **Sex** |
| practice | 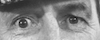 |  | jealous | **panicked** | arrogant | hateful | White | M |
| 1 | 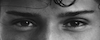 |  | **playful** | comforting | irritated | bored | White | M |
| 2 | 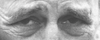 |  | terrified | **upset** | arrogant | annoyed | White | M |
| 3 | 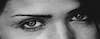 |  | joking | flustered | **desire** | convinced | White | F |
| 4 | 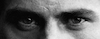 |  | joking | **insisting** | amused | relaxed | White | M |
| 5 | 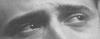 |  | irritated | sarcastic | **worried** | friendly | White | M |
| 6 | 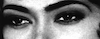 |  | aghast | **fantasizing** | impatient | alarmed | White | F |
| 7 | 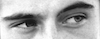 |  | apologetic | friendly | **uneasy** | dispirited | White | M |
| 8 | 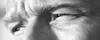 | yes | **despondent** | relieved | shy | excited | White | M |
| 9 | 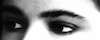 | yes | annoyed | hostile | horrified | **preoccupied** | White | F |
| 10 | 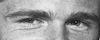 |  | **cautious** | insisting | bored | aghast | White | M |
| 11 | 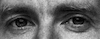 |  | terrified | amused | **regretful** | flirtatious | White | M |
| 12 | 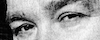 | yes | indifferent | embarrassed | **sceptical** | dispirited | White | M |
| 13 | 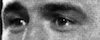 |  | decisive | **anticipating** | threatening | shy | White | M |
| 14 | 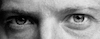 | yes | irritated | disappointed | depressed | **accusing** | White | M |
| 15 | 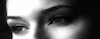 | yes | **contemplative** | flustered | encouraging | amused | White | F |
| 16 | 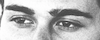 |  | irritated | **thoughtful** | encouraging | sympathetic | White | M |
| 17 | 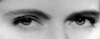 |  | **doubtful** | affectionate | playful | aghast | White | F |
| 18 | 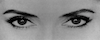 |  | **decisive** | amused | aghast | bored | White | F |
| 19 | 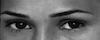 | yes | arrogant | grateful | sarcastic | **tentative** | White | F |
| 20 | 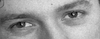 |  | dominant | **friendly** | guilty | horrified | White | M |
| 21 | 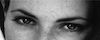 |  | embarrassed | **fantasizing** | confused | panicked | White | F |
| 22 | 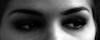 | yes | **preoccupied** | grateful | insisting | imploring | White | F |
| 23 | 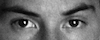 |  | contented | apologetic | **defiant** | curious | White | M |
| 24 | 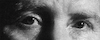 | yes | **pensive** | irritated | excited | hostile | White | M |
| 25 | 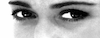 |  | panicked | incredulous | despondent | **interested** | White | F |
| 26 | 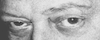 |  | alarmed | shy | **hostile** | anxious | White | M |
| 27 | 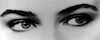 |  | joking | **cautious** | arrogant | reassuring | White | F |
| 28 | 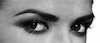 |  | **interested** | joking | affectionate | contented | White | F |
| 29 | 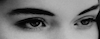 |  | impatient | aghast | irritated | **reflective** | White | F |
| 30 | 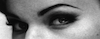 |  | grateful | **flirtatious** | hostile | disappointed | White | F |
| 31 | 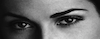 |  | ashamed | **confident** | joking | dispirited | White | F |
| 32 | 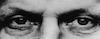 | yes | **serious** | ashamed | bewildered | alarmed | White | M |
| 33 | 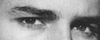 |  | embarrassed | guilty | fantasizing | **concerned** | White | M |
| 34 | 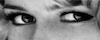 |  | aghast | baffled | **distrustful** | terrified | White | F |
| 35 | 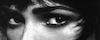 |  | puzzled | **nervous** | insisting | contemplative | White | F |
| 36 | 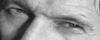 | yes | ashamed | nervous | **suspicious** | indecisive | White | M |

**Supplemental Table 2. Participant complaints about the Reading the Mind in the Eyes Test.** Comments were collected as feedback from TestMyBrain.org upon completion of the RMET.

| RMET Participant Comments | |
| --- | --- |
| **Racial homogeneity** | "The mind in the eyes was a bit challenging, and [I] am not sure if this could be because it was all white faces, and I could probably not relate easily with the expressions!"  “This patient shared feedback about diversity concerns. He had asked during enrollment and his initial check-in about the importance of diversity in the study (meaning, does the study value diversity?) and was assured both times that this was the case. … There was not a single image of a person of color, which can feel discouraging/unwelcoming and he is now feeling ambivalent about continuing in the study.” |
| **Gender stereotyping** | "[It] was a good test but I thought that the emotions through eyes text was sexist as any emotion to do with desire or fear I thought was a woman and any emotion which was to do with power or kindness was from a man.”  "As I previously stated, your eyes/emotion test is total sexist [********]. Not all women wear a [****]-ton of makeup and like to be used for male fantasy emotions like "desire", etc. Why don't you hire some female researchers. Shame on you and your male sexist bias. Shame.”  "I thought the photos with the emotions were rather gender biased i.e reflected media stereotypes" |
